# A practical approach to low protein diets for patients with chronic kidney disease in Cameroon

**DOI:** 10.1186/s12882-016-0340-5

**Published:** 2016-09-07

**Authors:** Gloria Enow Ashuntantang, Hermine Fouda, Francois Folefack Kaze, Marie-Patrice Halle, Crista Tabi-Arrey, Magloire Biwole-Sida

**Affiliations:** 1 Yaounde General Hospital & Faculty of Medicine and Biomedical Sciences, University of Yaounde I, Yaounde, Cameroon; 2 Douala General Hospital & Faculty of Medicine and Biomedical Sciences, University of Yaounde I, Yaounde, Cameroon; 3 University Teaching Hospital Yaounde & Faculty of Medicine and Biomedical Sciences, University of Yaounde I, Yaounde, Cameroon; 4 Douala General Hospital & Faculty of Medicine and Pharmaceutical Sciences, University of Douala, Douala, Cameroon; 5 Yaounde Central Hospital, Yaounde, Cameroon; 6 Faculty of Medicine and Biomedical Sciences, University of Yaounde I, Yaounde, Cameroon

**Keywords:** Low protein diets, Chronic kidney disease, Cameroon, Africa

## Abstract

Cameroon is a low–middle income country with a rich diversity of culture and cuisine. Chronic kidney disease (CKD) is common in Cameroon and over 80 % of patients present late for care, precluding the use of therapies such as low protein diets (LPDs) that slow its progression. Moreover, the prescription of LPDs is challenging in Cameroon because dieticians are scarce, there are no renal dieticians, and people often have to fund their own healthcare. The few nephrologists that provide care for CKD patients have limited expertise in LPD design. Therefore, only moderate LPDs of 0.6 g protein per kg bodyweight per day, or relatively mild LPDs of 0.7–0.8 g protein per kg bodyweight per day are prescribed. The moderate LPD is prescribed to patients with stage 3 or 4 CKD with non-nephrotic proteinuria, no evidence of malnutrition and no interrcurrent acute illnesses. The mild LPD is prescribed to patients with stage 3 or 4 CKD with nephrotic proteinuria, non-symptomatic stage 5 CKD patients or stage 5 CKD patients on non-dialysis treatment. In the absence of local sources of amino and keto acid supplements, traditional mixed LPDs are used. For patients with limited and sporadic access to animal proteins, the prescribed LPDs do not restrict vegetable proteins, but limit intake of animal proteins (when available) to 70 % of total daily protein intake. For those with better access to animal proteins, the prescribed LPDs limit intake of animal proteins to 50–70 % of total daily protein intake, depending on their meal plan. Images of 100 g portions of meat, fish and readily available composite meals serve as visual guides of quantities for patients. Nutritional status is assessed before LPD prescription and during follow up using a subjective global assessment and serum albumin. In conclusion, LPDs are underutilised and challenging to prescribe in Cameroon because of weakness in the health system, the rarity of dieticians, a wide diversity of dietary habits, the limited nutritional expertise of nephrologists and the unavailability of amino and keto acid supplements.

## Background

Cameroon is a low–middle income sub-Saharan African country with a population of 22.5 million and a gross domestic product of 34 billion US dollars [1]. Life expectancy at birth is 58 years, infant mortality is 88 per 1000 births and maternal mortality is 590 per 100,000 live births. Health expenditure constitutes 5.1 % of the gross domestic product, and private health expenditure accounts for 67.6 % of this expenditure, of which 94.2 % is funded by the patients themselves [2]. About 46 % of the population have access to improved sanitation and 47 % live in rural areas. There is a huge deficit in manpower in the healthcare system with less than one physician and 4.4 nurses per 10,000 inhabitants [2].

While the true prevalence of chronic kidney disease (CKD) in Cameroon is unknown, CKD is common, with a prevalence of 13.2 % reported in one region [3]. There has also been a gradual increase in the number of Cameroonians receiving long-term haemodialysis [4, 5]. Although several measures are known to slow the progression of CKD, such as low protein diets (LPDs), the late presentation to nephrologists of patients with CKD often precludes their use. We have previously reported that 55.7 % of patients with CKD are seen for the first time in our clinic at stage 5, with one third of them requiring emergency dialysis [6].

## Food and dietary habits in Cameroon

The habitual diet of Cameroonians provides a daily energy intake of 14.1 -18 MJ with only 8.9–10.4 % of total energy intake from proteins compared to 45.3–48.7 % from carbohydrates and 42.0–43.8 % from fats [7]. In Cameroon, the daily protein intake of adult women has been estimated at 88 g in rural residents and 86 g in urban residents. In adult men, the daily protein intake values are higher at 94.5 g in rural residents and 100.1 g in urban residents [7]. These values probably represent a daily protein intake of more than 1 g per kg bodyweight. In rural areas, dietary proteins are mainly derived from leafy vegetables, legumes, grains, seeds and mushrooms, while in urban areas, animal proteins account for a significant part of protein intake [7–11]. Several studies have confirmed the high protein value of most of the indigenous leafy vegetables, legumes, grains and spices used for food in Cameroon [12–20]. Traditional home meals using staple foods are the mainstay of diets in rural areas, while Western-style foods and takeaway fast foods are becoming increasingly common in urban areas [7, 21]. Indigenous Cameroonian meals are usually prepared as a composite mixture of different foods served as sauces or soups alongside a starchy food [7, 10, 16, 18, 21, 22], though occasionally the starchy food is included in the sauce and served as a single dish. These sauces usually contain leafy vegetables, legumes and seeds with meat or fish added depending on affordability, availability and cultural habits. Starchy foods commonly consumed in Cameroon include corn, millet, sorghum, cassava (tubers of *Manihot utilissima*), yam (tubers of *Dioscorea sp.*), cocoyam (tubers of *Xanthosoma sp.*), potatoes, plantains, unripe bananas and rice [7, 8, 10, 11, 16, 21, 23]. Examples of common composite meals and their compositions are shown in Table 1. Meal frequency depends on affordability, usually varying from twice daily in rural areas to thrice daily in urban areas. Meals consumed by rural populations and low-income urban populations typically include breakfast (usually left-overs from the previous supper) and supper. Breakfasts consumed by urban and semi-urban populations typically consist of bread and fried mixtures of wheat flour, cassava or corn dough (locally called puff-puff, acra or beignets) eaten with a bean sauce, pap (hot cereal composed of fermented corn, sorghum or millet flour, mixed with water, sugar and lemon), meatballs or an omelette [7, 21]. Breakfasts consumed by the lower and lower–middle class in semi-urban and urban areas are often purchased as fast foods from mobile or roadside restaurants [24].

**Table 1:** A selection of some common composite meals in Cameroon

Meals	Compositions
Okra pod soup	A soup that is prepared with fresh okra (*Abelmoschus esculentus*), dried fish or fresh beef, tomatoes, spices, oil. Ground egusi or peanuts may be added. Usually eaten with garri, corn or cassava fufu
Bean stew	A stew made of dried beans, tomatoes, crayfish, onions and palm oil.
Sanga	A dish of fresh corn boiled with cassava, huckleberry or pumpkin leaves in palm nut pulp. It is eaten as a meal.
Corn chaff	A sauce of boiled dry corn grains (*Zea mays* L) and dry grains of beans (*Phaseolus vulgaris*), cooked in palm oil (*Elaeis guinensis*), with limestone, Maggi cube, kitchen salt, onions, ginger, garlic, and crayfish as main ingredients.
Bobolo or miondo	A paste of fermented cassava (*Manihot utilissima*) wrapped in leaves and steam boiled.
Cassava fufu	A dish of fermented cassava flour cooked into a paste and served with different soups.
Corn fufu	A dish made of dried ground corn and cooked into a paste. It is eaten with okra and njama njama soups.
Garri	A fine to coarse granular flour of varying texture made from cassava which is cleaned after harvesting, grated, water and starch squeezed out of it, left to ferment and then fried either in palm oil or without palm oil. It can be eaten as a snack in cold water with sugar added, or cooked in hot water to make a dough-like paste eaten with a sauce.
Pap	A dish made of hot cereal, fermented corn, sorghum or millet flours and mixed with water, sugar and lemon. It is eaten with bread or Beignet for breakfast. It is also a weaning food.
Beignets	Semi-liquid paste of wheat, cassava, and corn or bean flour sweetened with either sugar or banana or salted the paste is deep fried in cooking oil in round shapes. Also known as *puff -puff* or *acra* in the English–speaking regions
Njama njama	A dish made of Huckleberry leaves cooked and sautéed in palm oil. It is eaten with corn fufu.

Uncooked foods are sold mainly in markets that open daily in semi-urban and urban areas and periodically in rural areas. With the exception of beef, pork and fish, which are sold per weight, other indigenous foodstuffs are neither weighed nor labelled. Legumes and cereals are quantified for sale using standard containers for measurement such as 150 ml plastic bowls or empty cans of tomato paste or concentrated milk that are widely available.

## Dietary prescription

Dieticians are scarce in Cameroon and when available are not dedicated to renal patients. Dietary prescriptions for the majority of CKD patients are therefore made by nephrologists who usually lack expertise in renal nutrition and who are often responsible for the care of a large number of patients. Only two of the nine hospitals in Cameroon with renal services have a dietician, and these dieticians service the entire hospital patient population. In a recent survey in our unit, less than a quarter of our patients in the pre-dialysis clinic had consulted a dietician about a renal diet (unpublished data). Nephrologists in Cameroon have thus learnt how to design diets for CKD patients during their clinical practice.

## LPDs in Cameroon

The prescription and design of LPDs in Cameroon are challenging because of resource limitations, a shortage of healthcare personnel and protean dietary practices. In our practice, only moderate LPDs of 0.6 g protein per kg bodyweight per day (moderate LPD) and relatively mild LPDs of 0.7–0.8 g protein per kg bodyweight per day (mild LPD) are prescribed for CKD patients. Several types of LPD have been well-described including the traditional LPD, vegan LPD, vegan LPD supplemented with amino and keto acids, LPD with protein-free food and a very low protein diet of 0.3 g protein per kg bodyweight per day [25]. Only the traditional LPD with mixed protein intake is feasible in Cameroon. Amino and keto acid supplements are not available locally and their cost precludes importation for the majority of patients. The moderate LPD is prescribed to patients with stage 3 or 4 CKD who have non-nephrotic proteinuria, no interrcurrent acute illnesses and no clinical evidence of malnutrition. The mild LPD is prescribed to patients with stage 3 or 4 CKD with nephrotic proteinuria but no signs of malnutrition, CKD with no uremic signs or symptoms and stage 5 CKD on non-dialysis treatment. In the absence of pre-weighed foods and meals, we have devised simple ways of enabling patients to estimate protein quantities, such as the distribution of photographs of 100 g portions of meat; fish and chicken to illustrate quantities. We have learnt, for instance, that the drumstick of an average-sized chicken weighs approximately 200 g, and an average egg provides about 6 g of protein. We have also found published data on the protein content of composite meals similar to those sold in roadside restaurants to be very useful for diet design [11, 16, 18, 21]. For instance, data on corn chaff (corn, beans, palm oil, crayfish, salt, Maggi cube), a composite meal commonly sold in roadside restaurants, reveal that it contains 11.7 g protein per100 g and that a serving at these restaurants typically weighs about 640 g [18].

We do not limit intakes of vegetables, legumes or cereals for patients in rural areas for whom these foods constitute the main source of protein. In this group of patients, animal proteins are only eaten sporadically either during ceremonies or when a wild animal (bush meat) is killed during hunting or incidentally. We do not restrict this occasional animal protein intake either, provided that the prescribed total daily protein intake is not exceeded. For patients who go for several months with no access to animal proteins, we encourage the intake of soya bean at least once a week if available locally. For patients whose habitual diet is rich in animal proteins, we prescribe animal proteins to constitute 50–70 % of the total protein intake, with vegetables, legumes and cereals providing the remainder. This quantity of animal proteins can be consumed daily or on set days. For those patients consuming animal proteins daily, the animal proteins constitute 50–60 % of the total daily protein intake. For those consuming animal proteins on set days, animal proteins constitute 60–70 % of total daily protein intake on these days and 0 % of total daily protein intake on the other days. Most patients choose 2–3 animal protein free days per week. An example of a 7-day menu for a traditional moderate LPD prescribed to a middle-class Cameroonian residing in an urban area with two animal protein free days is presented in Table 2. Energy requirements and other specific dietary restrictions associated with CKD are taken into consideration when designing the diet. In order to promote adherence to the prescribed diet, we explain the diet to the patients as well as those who prepare their meals (usually the female relatives of male patients, as is the tradition in most indigenous Cameroonian families). Dietary habits, meal frequencies and economic status are also important factors we consider when designing a diet.

**Table 2:** A 7-day 0.6 LPD menu for a 60 kg urban patient in Cameroon

Meal	Day1	Day 2	Day 3	Day 4	Day 5	Day 6	Day 7
Breakfast	**Beignets** and, beans stew	1 piece of meat (100 g) in s cabbage stew with, onions Boiled Irish potatoes or **bobolo**	1 slice of fresh fish (50 g) in stewed leafy vegetables with lots of onions Boiled Sweet Potatoes	25 g of minced meat with steamed mixed vegetables and diced boiled Irish potato in salad	1/4 medium avocado, vegetable salad with low fat salad dressing Bread Tea with sugar	Corn or millet pap, bread or beignets	1 slice of fish (50 g) in soup Boiled Plantain or **bobolo**
Lunch	Stewed local leafy vegetables with soyabean or groundnut paste. Boiled **cocoyam**	11/2 slices of fresh fish (70 g) with mixed vegetables in stir fry Boiled yam	2 tablespoons of ground crayfish in stewed local leafy vegetables with lots of onions Boiled **Irish Potatoes**	Mixed local vegetables in or tomato sauce 300 g (15 heaped tablespoons) of boiled white rice	01 drumstick of skinned chicken in stewed cabbage with lots of onions, celery and leeks Boiled plantain	Cassava leaves and palm nut pulp in soup served with boiled cassava OR **Sanga**	1 slice of steamed fish (50 g) in garden eggplant leaves in stir fry Boiled plantains
Supper	Vegetable salad and bread Lemon grass tea	15 g of smoked fish in **okra** soup served with garri	2 tablespoons of soybean paste in stewed cabbage Boiled unripe bananas	1 medium chicken drumstick (100 g), in soup Boiled Irish potatoes	**Egusi** or groundnut okra soup with crayfish **Corn fufu**	Mixed vegetables in tomato sauce pasta or rice (boiled)	A slice of fresh fish (50 g), flaked with melon leaves in stir-fry Boiled **sweet potatoes**

**Beignets**: deep fried semi-liquid paste of wheat, cassava, and corn or beans flour sweetened with either sugar or banana or salted the; **Bobolo**: steam boiled paste of fermented cassava (*Manihot utilissima* roots); **Cocoyam**: tuber of *Xanthosoma sp.*; **Corn fufu**: cooked paste of dried ground corn; **Garri**: A fine to coarse granular flour made from fermented cassava fried either in palm oil or without palm oil; **Irish potatoes**: *Solanum tubrosum*; **Okra**: *Abelmoschus esculentus*; **Pap**: a dish made of hot cereal of fermented corn, sorghum or millet flours and mixed with water, sugar and lemon; **Sanga**: dish of fresh corn boiled with cassava, huckleberry or pumpkin leaves in palm nut pulp; **Sweet potatoes**: tuber of *Ipomoea batatas*,; **Yam**: tuber of *Dioscorea sp*., **Egusi**: ground melon seeds (*Cucumeropsis mannii*)

## Nutritional monitoring of patients

The reliance on patient funds for healthcare means that we do not request additional clinic visits for patients on LPDs or use multiple Para clinical tests to assess or monitor their nutritional status, because they are generally unaffordable. However, before prescribing the diet, we do evaluate the body mass index of patients without oedema; and perform a subjective global assessment of nutritional status using a validated questionnaire. Para clinical assessments are limited to a serum albumin assay, which may be requested if the patient can afford it. This evaluation is performed at each follow-up visit, with the exception of serum albumin which is requested 6-monthly. We have also used haemoglobin and serum phosphorus and cholesterol levels as indicators of nutritional status, measured as part of routine patient care. Adherence to LPD is poor in our practice. Only 36 % of 28 patients prescribed a traditional 0.6–0.8 LPD in our outpatient clinic were adherent (Unpublished data). In our experience, assessments of patient nutritional status during their routine follow-up visits are sufficient and do not significantly increase consultation time.

## Conclusions

In conclusion, the use of LPDs for CKD patients is challenging in Cameroon. There are no renal dieticians, and the nephrologists who play this role have limited nutritional expertise. Furthermore, the lack of amino and keto acid supplements as well as the composite nature of meals in Cameroon limit the type of LPD that can be prescribed. The high rate of late presentation for care observed in CKD patients and the high personal cost of healthcare precludes the use of this therapy for many patients. Several strategies may increase the use of LPDs in CKD patients in Cameroon, such as improving the knowledge and practice of nephrologists and nurses in nutrition and making amino and keto acid supplements accessible to patients.

## Abbreviations

CKD: Chronic kidney disease
LPD: Low protein diet
